# Geographies of education, volunteering and the lifecourse: the Woodcraft Folk in Britain (1925–75)

**DOI:** 10.1177/1474474014536855

**Published:** 2016-01

**Authors:** Sarah Mills

**Affiliations:** Loughborough University, UK

**Keywords:** informal education, lifecourse, volunteering, Woodcraft Folk, youth

## Abstract

This article extends the current scholarly focus within the geographies of education and the geographies of children, youth and families through an original examination of the Woodcraft Folk – a British youth organization founded in 1925 that aimed to create a world built on equality, friendship and peace. This article illustrates how voluntary uniformed youth organizations had a much wider spatial remit and more complex institutional geographies than have been hitherto acknowledged, with their active involvement in the training of adults (namely *parents* and *volunteers*) as well as the education of *children and young people.* Drawing on archival research and a range of sources, the article explores the Woodcraft Folk’s philosophies and political activities across its first 50 years, and in doing so, makes two central academic contributions to the discipline. First, the article provides a timely focus on *training* and its analytical purchase for geographers as part of a growing body of work on the geographies of education. Second, the article shows how geographers can account for both children *and* adults’ geographies in institutional spaces, in this case through mapping out the enlivened historical geographies of voluntarism across the lifecourse. This article demonstrates the complex and often fluid relationship between formal and informal education, as well as the important connections between parenting and volunteering. Overall, the article reflects on the subsequent challenges and opportunities for researchers concerned with debates on education, youth and volunteering within geography and beyond.

‘The specific business of woodcraft is educational’

‘Shada’, Woodcraft Folk Elder, 1935

At the 10-year anniversary of the fledgling Woodcraft Folk – a British youth organization – ‘Shada’ rebuffed comments from other committee members that its purpose was *political* by stressing that *education* was its primary objective. The Woodcraft Folk still exists today in the United Kingdom, positioning itself as a space of informal education for 6–20-year-olds that is committed to issues of social justice, pacifism and the principles of cooperation, attributes that have distinguished it from other uniformed youth movements since its formation by Leslie Paul in 1925. Whilst in their name and target audience, British youth movements that emerged in the late 19th and early 20th century were focused on the education of *children and young people*, this article’s central argument is that they were also actively involved in the education – or as I will show *training* – of adults, namely *parents* and *volunteers*. Through an examination of the Woodcraft Folk’s philosophies and activities across its first 50 years (1925–75), I show that voluntary uniformed youth organizations had a much wider spatial remit and more complex institutional geographies of education across the lifecourse than have been hitherto acknowledged. Indeed, literature on British youth movements has not recognized the wider educational programme, activities or lobbying of such voluntary organizations and the ‘specific [educational] business’ that Shada alluded to in 1935.^1^ Drawing on archival research, this article builds on earlier work on the cultural-historical geographies of youth and citizenship education^2^ but importantly extends the current scholarly focus within the geographies of education^3^ and geographies of children, youth and families^4^ to make two central academic contributions to the discipline more broadly.

First, the article provides a timely focus on ‘training’ and its analytical purchase for geographers through an original examination of the relationship between formal and informal learning spaces. Although this article draws upon historical data from 1925–75, I contend that the need to consider training is vitally important in understanding a range of historical and contemporary sites and settings. A variety of schemes and organizations (for adults *and* young people) purport that their remit is training, rather than education. Training suggests a particular type of learning: often skills-based, staged, repeated, refined, observed, assessed and perhaps most significantly, is often *re-learned* – in some cases, after a long period of time and usually with varied age dynamics. It suggests perceived sets of knowledge that are seen to be needed by either young people and/or adults for various vocations or activities, including – as this article shows – training ‘for life’ and ‘the future’. Training, across a variety of spaces, therefore poses questions about the very definitions and nature of work, education, care and everyday life.^5^ In exploring these shifts over time through a cultural-historical approach, this article advances current debates in the growing field of geographies of education where the existing focus has tended to be on contemporary learning spaces and more firmly rooted in social geography. Indeed, although the boundaries and relationships between social and cultural geography are ever-changing^6^ and some work locates itself within social *and* cultural geographies of education,^7^ this article does contribute a significant cultural dimension to research on the geographies of education and learning. There has been some previous work on education and learning in a more cultural-historical vein, and this has focused on schooling and citizenship^8^ as well as examining the role of educational sites and activities in shaping wider moral geographies of gender, class and nationhood.^9^ Here, this article significantly develops some of these themes around education and citizenship, but adds a new focus on *training* in response to Holloway and Jöns recent call for approaches that attend ‘to the relationship between formal and informal spaces of education and learning’.^10^ The paper illustrates through the example of the Woodcraft Folk how the boundaries between some of the spaces and practices of informal and formal education are not static, but complex and fluid. Furthermore, it shows that within this organization there were concerted efforts to educate (or train) *parents* and *volunteers* in addition to young people.

The second academic contribution of this article is therefore in demonstrating how geographers can account for both children *and* adults’ geographies in institutional spaces, in this case through mapping out the enlivened historical geographies of voluntarism across the *lifecourse.* This article contends that British youth movements such as the Woodcraft Folk trained and enfolded people of all ages into their activities: children, parents and adult volunteers. Furthermore, I show how this institutional space was ‘accomplished’^11^ through familial and community-based ideologies and structures, bringing together work and synergies between the geographies of voluntarism and the geographies of parenting that have been marginalized in the literature thus far. The article makes a specific argument about the complex dynamics of age within this institutional space, in particular surrounding youth volunteering, and therefore attends to Hopkins and Pain’s call for geographers to move away from the ‘social-chronological margins’ and towards more relational geographies of age^12^ that tease out some inter-generational^13^ connections – in this case between families, folk ‘elders’, adult volunteers and young people.

The following sections first position this article within the relevant bodies of literature and academic debates, as well as discussing the methods and sources upon which this research is based. I then outline the educational programme of the Woodcraft Folk aimed to train young people to ground the following discussion about the ‘training’ of parents and adult volunteers, before offering some conclusions on the wider implications and relevance of this study.

## Geographies of education, volunteering and the lifecourse

### Geographies of (in)formal education, learning and training

In recent years, research on the geographies of education and learning has brought together studies from a range of diverse sub-disciplines – although primarily from social geography – that ‘consider the importance of spatiality in the production, consumption and implications of formal education systems from pre-school to tertiary education and of informal learning environments in homes, neighbourhoods, community organisations and workspaces’.^14^ One question this article poses is what might a focus on training – as a specific type of pedagogical process – give researchers, or challenge them to consider, in relation to debates on education and learning? My argument here is for training to be more fully considered as part of the growing and vibrant field of geographies of education and with this, to make some useful crossovers and connections to geographical research on voluntarism,^15^ parenting^16^ and the lifecourse.^17^ As a type of organizational activity, training is often used as a synonym for education or learning, usually relating to notions of skill, practice and continual improvement, as well as re-inforced, refined or re-learned knowledge. There have been a few important but relatively isolated studies of training by geographers in terms of neoliberal work transitions and re-entry to the labour market,^18^ as well as research on wider economic and gendered geographies of skills-based training and ‘lifelong’ learning.^19^ These studies are increasingly relevant as the politics of workfare continue to intensify; for example, the recent furore over the UK government’s training programme for jobseekers where unpaid work was pitched as ‘experience’, ‘volunteering’ and ‘on-the-job training’.^20^ Here, I show that a geographical approach could be useful in furthering our understanding of diverse training spaces for adults *and* young people, beyond workfare contexts. In this study, I argue that as a youth organization in civil society, the Woodcraft Folk operated as an ‘in-between’ space of training (after Philo et al.^21^) with a complex and sometimes contradictory position on its educational philosophies and practices.

Whilst principles of training underlie many of the pedagogies and habits of formal schooling,^22^ training is often used to distinguish between formal and informal education. For example, in the United Kingdom, ‘citizenship *education*’ has been delivered in schools through formal lessons and taught classes^23^ whilst ‘citizenship *training*’ was espoused by a range of voluntary youth organizations across the 20th century.^24^ Ultimately the Woodcraft Folk operated a youth citizenship training programme on a voluntary basis and positioned themselves as the antithesis to school, drawing upon the varied, spontaneous, everyday processes and practices of *informal* learning that can take place in a range of settings.^25^ However, at the same time, this article shows how the organization utilized techniques and materialities associated with formal classrooms for training both children and adult volunteers, drawing on notions of proficiency and observed assessment. Despite this contradiction, the Folk continually advocated the principles of informal learning and called for a series of reforms in relation to state education. This article therefore illustrates some of the connections and interplay between formal and informal education through an analysis of this organization across a number of decades. In doing so, the article tentatively provides one way through which the geographies of education could capture other, diverse spaces of training over time and utilize a cultural-historical approach. Furthermore, it also pushes at some related debates on the geographies of voluntarism and the geographies of children, youth and families.

### Geographies of voluntarism

Christine Milligan’s mapping of the geographies of voluntarism in 2007 captured social and economic research on volunteering and the voluntary sector.^26^ Since then, the field has steadily grown; for example, through important studies on global youth volunteering^27^ and the relationship between volunteering, higher education and employment.^28^ This present article is partly inspired by Fiona Smith et al.’s impassioned call for ‘enlivened’ geographies of volunteering that ‘considers voluntary action as a set of situated, emotional and embodied practices’.^29^ Whereas Smith et al. discuss contemporary volunteering practices across different social and welfare settings, here I present an enlivened *historical* geography of voluntarism. Historians of volunteering and social action have usefully traced the role of the voluntary sector in civil society and wider processes of state formation,^30^ but this article brings an important focus on the *training* of volunteers over time, in this case as part of a youth organization, to illustrate the central role adult volunteers played in the production, maintenance and negotiation of such spaces. Furthermore, the article highlights some unique approaches to age by the Woodcraft Folk through open notions of ‘youth’ volunteering that highlight the complex boundaries between childhood and adulthood.^31^

The article also highlights the significant connection between volunteering and parenting that has yet to be fully explored by geographers. Whilst important studies have examined parenting practices in relation to childcare and employment^32^ and gendered emotional identities,^33^ I contend that geographers are also well placed to study some of the connections between parenting and volunteering. There has always been a strong historical connection between the two practices. For example, the now international playgroup movement was founded after a letter from a mother concerned over the lack of provision for under 5’s appeared in *The Guardian* in 1961 outlining a ‘do-it-yourself’ nursery school campaign.^34^ In contemporary contexts, geographers have highlighted the informal volunteering practices of mothers in studies on community participation,^35^ as well as the propensity for some parents to volunteer in order to enable their children to access youth organizations and clubs.^36^ This present article shows how, over a number of decades, parents were enfolded into the everyday rhythms of the Woodcraft Folk, often as volunteers, but also at home and through other meeting spaces as part of the wider organizational family. In doing so, this article illustrates the possibilities for further enlivened geographies of voluntarism that connect with wider research on the geographies of children, youth and families.

### Geographies of age and the lifecourse

The final series of academic debates and literature this article speaks to surround age and the lifecourse. In response to Hopkins and Pain’s call to think relationally about age,^37^ this article brings together a focus on children, young people, *and* adults in the context of this case-study, rather than solely on the extremes of chronological age-groups. As outlined in the introduction, the article’s central argument is that the Folk was not only designed to attract and educate (train) children, but also embraced adults and parents into its wider pedagogical practices. Elsewhere, I have argued that ‘youth movements utilise and prioritise the liminal period of youth as a critical and necessary stage in the lifecourse in which to harness and secure an individual’s (future) potential and political capital for their cause(s)’.^38^ However, here I wish to extend this argument by demonstrating that these spaces also positioned adults as in need of learning and created parallel training systems for adults as unpaid volunteers, as well as extending their moral compass into commentaries on, and instructions for, parents. Here, I draw upon ideas of the lifecourse to analyse the dynamic ways in which the Woodcraft Folk thought about age – for example, how training classes for adult volunteers were completed by some young teenagers, and how the organization considered the most effective adult volunteers to be young ‘at heart’ or ‘in spirit’. In valuing a sense of youthfulness, I argue that the Folk continued to draw on a series of idealized constructions of childhood as desired attributes in their responsible adult leaders.

This article draws on a range of Woodcraft Folk material published between 1925 and 1975, including yearbooks, annual reports, correspondence, committee minutes, membership records, training materials, posters, policy publications and other ephemera. This material was accessed during fieldwork at the Youth Movement Archive housed at the London School of Economics (LSE) Library in London, UK (original material is referenced as WF/reference in the notes), with subsequent coding and analysis in relation to the research themes. The first 50 years of the Folk was chosen due to the establishment and maintenance of its training programme during this time, although there are some interesting parallels with the contemporary period that are hinted at throughout this paper. There was clearly a wide range of social, economic and political changes in British society between 1925-1975. However, it is not the purpose of this article to use the Woodcraft Folk to make a commentary on these shifts or provide a political historiography. Rather, I draw on historical data to illustrate this article’s wider argument about the geographies of education, volunteering and the lifecourse through focusing on this one organization across a sustained period of time.

## Training youth: ‘that when I am older, I may take my place’

The Woodcraft Folk was founded by a young person. Leslie Paul was 20 years old when he started the radical youth organization in 1925 and, as ‘Little Otter’, would shape the movement in its formative years. He described that the organization, ‘first considered an eccentricity of importance only to my adolescent self, has become an important national auxiliary’.^39^ The Folk has a fascinating genealogy tied up with a short-lived youth organization called the Kibbo Kift Kindred that originally broke away from the Boy Scout Movement after the First World War. The Folk also had a series of political wranglings with the Labour Party and Co-operative Movement as is strove to secure endorsements and financial support, although both these facets of its past have been charted by academic historians.^40^ Leslie Paul’s original vision was to create a world built on equality, friendship and peace, describing the Folk as standing for ‘world peace and co-operation, camping and handicrafts, mental and physical fitness’.^41^ Its primary audience was working-class British youth, encouraged to join local groups structured into age-based sections run by volunteers. There were no paid staff in the original organization, with groups meeting throughout the UK in community centres, school halls and sometimes in homes. Voluntary leaders would organize weekly evening meetings with a varied programme of arts, crafts, games and lessons in ‘folk culture’, as well as leading a few camps each year and other day-trips and outings.^42^ Other volunteers would operate at a regional and national level to coordinate finance, publicity and training, as outlined later in this paper. After the Second World War, there were some ‘organizers’ employed on sporadic and unstable contracts to coordinate national youth work, but these were isolated and instead it was through the energy of volunteers and young people that Leslie Paul began to coordinate what he termed a ‘powerful educational instrument’^43^ that grew from 70 young people in 1925 to 14,780 by 1975.^44^ In this section, I focus on the justification behind the Folk’s youth citizenship training programme in the context of this article’s wider argument.

**Figure 1.**
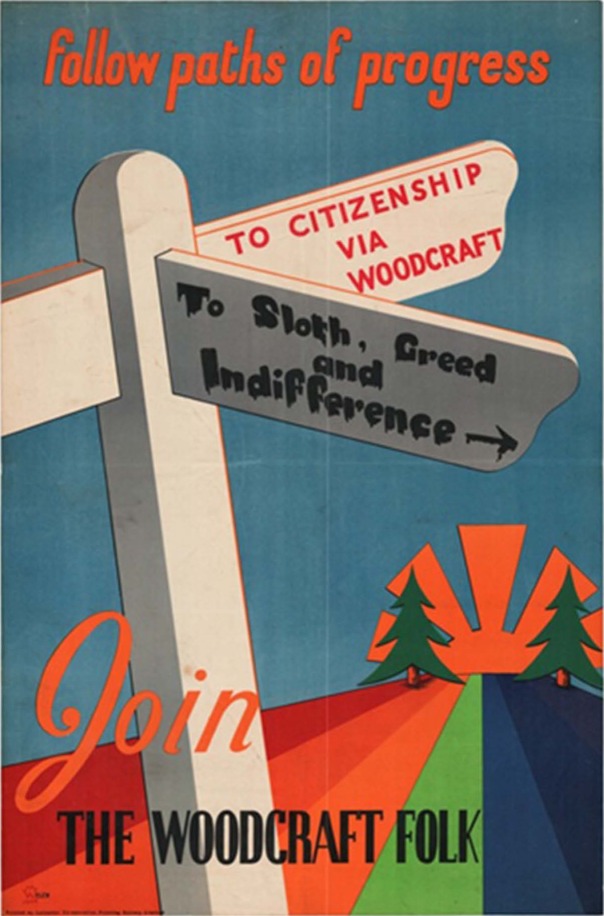
‘Follow Paths of Progress’ (YMA/WF/358/III). With kind permission from the Woodcraft Folk and the London School of Economics Library.

Most British youth movements constructed their archetypal ‘young citizen’ and translated a cluster of moral geographies around duty, nationhood and gender into a weekly, adult-led adventurous programme of activities for young people – the most popular being the uniformed Boy Scout and Girl Guide Movements. The Folk, whilst having radically different political, religious and gender-based ideologies to scouting or guiding – as both secular and co-educational – still embedded ideas of youth training, progress and development as part of their rationale and overarching philosophy. In the most basic sense, age was the first criteria for moving ‘up’ through the various sections from ‘elfins’ (7–10) to ‘pioneers’ (10–14) until a ‘kinsman or woman’ (14–20).^45^ However, more broadly, there was an overarching directional leaning towards future adulthood. For example, the organization often referred to their members as ‘citizens of tomorrow’^46^ and upon joining a local group, a young person made a declaration ‘1) to camp out and keep fit in mind and body 2) to work for world peace and co-operation 3) to understand the mysteries of nature and the history of the world, that *when I am older I may take my place* as an intelligent and useful member of mankind [sic]’.^47^ In an early poster, (See Figure 1) the organization clearly demarcated two ‘paths of progress’ that young people could follow:

Not only does this image position young people at a crossroads that they have to ‘arrive’ and progress to *somewhere*, but that they must make a decision to follow either the path to citizenship or the murky alternative of ‘sloth, greed and indifference’ off the beaten track. The poster suggests a clear cut moral landscape of two different journeys and two different destinations, but that the enlightened path involved *training*: ‘via woodcraft’ was the mechanism through which children would arrive at the desired location of citizenship.^48^ Further descriptions of the aims and rationale of the Woodcraft Folk evoke a type of neurological plasticity,^49^ with a set of reports and accounts from 1937 stating ‘[t]here is so much more to be done, so many millions of young and “plastic” minds awaiting the vivifying and rationalizing touch of our method, that our common aim must always be – better groups, more groups, and still more groups’.^50^ The Folk also positioned itself as an urgent and necessary space or stage in the lifecourse itself, stating that it could act ‘before the repressions of child life under capitalism have been intensified by industry and commerce into which children plunge after school, *before it is too late*’.^51^

In terms of the regular training the Folk provided to encourage young people to continue on the road to citizenship, the organization drew upon philosophies of informal or non-formal education. In part, the organization believed that they helped form ‘groups of children and young people *training themselves* towards the fulfilment of the ideals expressed in the Charter and Children’s declaration’.^52^ Indeed, they described their activities as ‘free in educational method’,^53^ that they believed children ‘learn by doing’^54^ and that their ‘education and . . . influences are applied through the interests – the voluntarily undertaken activities – of the child. We have an advantage the schools do not possess’.^55^ However, what I want to suggest here is that the Folk also used adult-child didactic teaching methods. For example, consider the formality of these instructions for a young person joining the Folk: ‘Learn by heart the creed and law – until you can stand up before your group or at campfire and RECITE correctly. Copy into your notebook – carefully – the DECLARATION as given above. THEN REPEAT it to yourself until you can recite it out aloud without looking at the words’.^56^ In many ways, this is ‘school-like’ – having notebooks and reciting verses to memory. Another example is how the *proficiency* of young people in certain activities was judged and tracked through individual record cards and the awarding of badges. I am not trying to suggest the Folk were not radical in their *content* – a quick look at some of the overtly political overtones in their badge programme, here seen on a membership record card from 1948 for ‘Wild Cat’ – a 10-year-old from Northern England – shows not only popular activities of athletics and crafting, but tellingly, knowledge about political theory and trade unions:

‘*Pioneer* (folk knowledge), *Supple Limb* (high jump), *Hiker* (map reading), *Backwoodsmanship, Lonecrafter, Festival Craft, Citizen, Athlete* (gymnastics) *Helper* (master of festival, propagandist, research worker), *Social History* (struggles for political freedom, comparative current political theory, exploration, agrarian revolution, local history)’.^57^

However, I do not believe the Folk were always as alternative in their training methods as they advocated. Indeed, the very fact they had badges during this period – one of the most popular elements of scouting and guiding – suggests the organization still felt they needed these formal tactile rewards for achieving proficient levels of aptitude and skill. This can be illustrated by the criteria for the early ‘World Citizen’ badge that included ‘draw a fairly accurate sketch map of the world from memory’ and ‘write a short essay explaining the objects and work of the League of Nations’,^58^ tasks that would have been assessed by an adult volunteer.

Overall, the programme of learning via badges within the Folk would have been cyclical and repeated each year with corresponding exams marked by an adult. In this respect, the Woodcraft Folk was a youth *training* scheme that espoused self-improvement and observed assessment, publishing educational materials that would ‘set and maintain a good standard of educational activity’,^59^ and in doing so, blurred the boundaries between informal and formal education.

## Training parents: records, reforms and rooftrees

In the next two sections, I demonstrate how the philosophies and activities of the Woodcraft Folk involved not just a programme of training for young people, but also for adults. These were, primarily, parents and volunteers, although it should be noted that sometimes parents were also volunteers and that other adults were ‘engaged’ in the Folk through wider political activism and fundraising. In terms of parents, there were both local and national strategies to enfold them into the very material fabric of the Folk. For example, at a local level, every young person’s record card (discussed in the previous section) also contained information about their parents in addition to badges achieved. For example, the record card of 14-year-old ‘SeaHawk’ from Dundee in 1949 quoted here shows information collected not just about him, but about his parents:

‘Age: 14

Folk name: SeaHawk

DOB: 05/04/1935

Co-operative member Y/N: N

Keen member of any other organizations: Youth Hostels Association

Education: St Patricks

Mother member of ___ guild ___ party: N/A

Parents no. and society [co-op]: 102154 St Mary’s

Parents supporters council attendance: limited’^60^

In noting that SeaHawk’s parents attendance at supporters council (discussed shortly) was ‘limited’, the card suggests that a series of judgements and observations were made by adult volunteers about individual parenting practices and related political affiliations. This could be seen as a subtle form of training parents, with clear appropriate or desired characteristics communicated to adult carers through the wider institutional spaces of this youth organization.

At a national level, there were also calls for parents to get involved and negotiate *school-based* events. For example, in the run-up to Empire Day in 1933 – a popular day of imperial vigour and celebration^61^ – Folk helpers across Britain were told that ‘Groups are asked to organize abstention of Pioneers from school on Empire Day . . . demand, *through parents*, that a Peace Day be held in schools instead of vulgar Empire Celebrations’.^62^ There is a sense here in which parents were seen as a ‘go-between’ for the Folk, able to move and mediate between school, home, and the Folk meeting place. In this respect, we can see how the programme the Folk provided outside the time-spaces of school was not detached or removed from formal learning spaces. The Folk also included parents in its wider educational and social activism, ‘demanding’ in 1930 that alongside ‘sweeping’ educational reforms, such as the raising of the school leaving age, children should be protected from ‘economic and social evils’ that included ‘incapable parents’.^63^ Whilst lobbying for a series of reforms in relation to formal, informal and alternative educational spaces, the Folk also advocated that ‘motherhood’ was protected and endowed. This perhaps reflects the organization’s own valued relationship with maternal symbolism; for example, the prominence of ‘mother nature’ in its programme.

**Figure 2.**
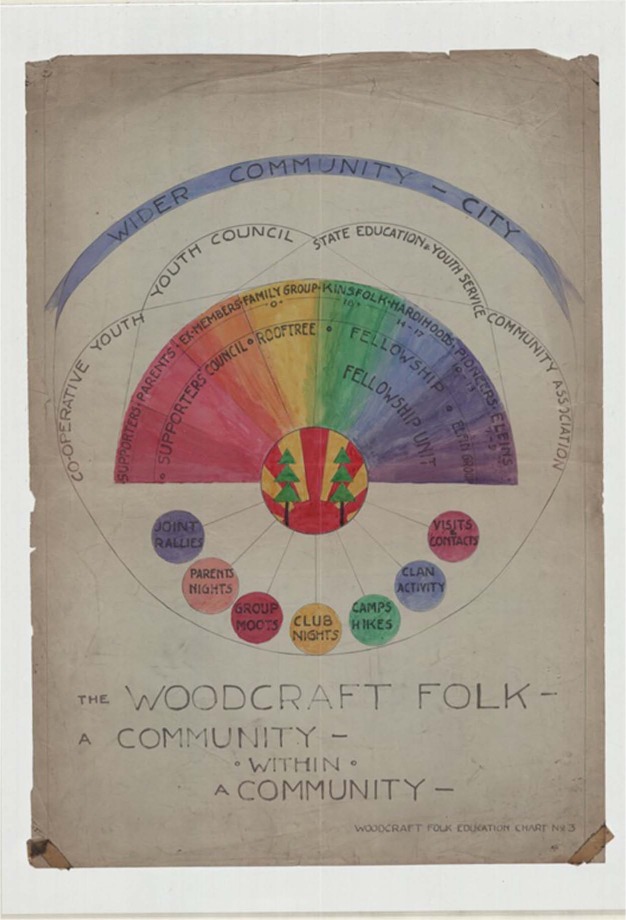
‘A Community Within a Community’ (YMA/WF/73/2I/8). With kind permission from the Woodcraft Folk and the London School of Economics Archives.

It is also important to highlight that in addition to these practical and political engagements surrounding parents and parenting, the Woodcraft Folk drew heavily upon notions of family in its wider scalar and spatial imaginaries, encapsulated here in Figure 2 (*c*.1950s–60s):

At the centre of this poster is the Woodcraft Folk’s symbol from which all its activities emanate in a series of circles and a coloured half-moon spectrum running from adults (left) to children (right). In the middle of the spectrum (yellow), ‘family groups’ called ‘rooftrees’ where children, young people and parents learned together, were an optional format for local groups, their very name suggesting rural connections to nature, shelter and comfort. In providing an overarching inter-generational canvas, the family was seen as the ideal unit through which the Folk could operate. Parents nights (bottom left, orange) were also encouraged, as were meetings of the ‘supporters’ council’ shown under parents, supporters and ex-members (red-orange) and mentioned earlier in relation to SeaHawk’s card. Above the coloured bands are wider regional and national structures such as ‘state education and youth service’. In this sense, the Folk saw themselves as connected to these other structures of formal education and informal youth work, as well as operating within scales such as the ‘wider community’ and ‘city’ appearing at the very top. Whilst it is too simplistic to suggest that this is how the Woodcraft Folk operated in practice, this image does encapsulate its ideal formation and spatialities – as ‘a community within a community’ – and with families at its core. As the organization stated in 1936, ‘the movement derives its strength from its members, and incidentally, the *families of members*’.^64^

It is therefore important to recognize the significance of families within the Folk’s ideology that operated in practice through a series of inter-generational voluntary time-spaces. Kraftl has recently highlighted the role of intra- and inter- generational relationships in contemporary (alternative) educational settings.^65^ Whilst there is a methodological challenge here to account for *past* relationships, emotions and friendships, there are clear signs that families (or as Kraftl has termed ‘family(-like) relations’) were important for supporting learning in the Woodcraft Folk. However, we know from record cards such as the one belonging to SeaHawk that parental support was not universal. In addition to these idealistic models, suggested reforms and record cards, the most powerful way for the Folk to ‘train’ parents was to enrol them as volunteers.

## Training volunteers: a ‘secure foundation’ or just ‘having a go’?

Woodcraft Folk helpers or leaders were technically defined as over 18; however, this section shows how in some cases much younger volunteers were part of the Folk’s operational apparatus. Overall, this section demonstrates how a wide-ranging national cohort of volunteers was mobilized to accomplish the Folk’s ‘institutional geographies’.^66^ Although informal adult training had existed locally since the organization’s foundation via a postal course of instruction with recommended reading and a returnable questionnaire, the Folk launched a formal training syllabus for leaders and helpers in 1938.^67^ The completion of this course by volunteers led to a Leader’s Diploma and certificate. The 25 lecture syllabus of the Folk’s adult training was based on a ‘textbook’ by Leslie Paul^68^ and included sessions on ‘the history of the Folk’, ‘psychology of the child’, ‘the child at school and home’ and ‘trade unions’. The topics covered not only mirrors some of the children’s badge programme discussed earlier, but also reflects growing interest in educational and psychological theory in the early 20th century;^69^ indeed, children are referred to as ‘our material’ throughout the training literature. To pass the course, volunteers were required to have 75 percent attendance at recognized classes, a minimum mark of 50 percent in a two-hour written short-answer exam paper, an inspection of their notebooks, and a one week training camp where the candidates ‘must satisfy the education committee’.^70^ If successful, volunteers also had an option to pursue an advanced diploma with further study. This perhaps confounds popular beliefs that voluntary organizations have always been desperate for support and therefore not enforced standards. Furthermore, I would argue that this system was used to standardize practice, recognize achievement and train leaders in required sets of knowledge. Indeed, despite advocating a less adversarial style of practice than scouting and other early British youth movements, the Folk still created a standard for volunteers to achieve in order to ‘apply [their] knowledge and ability . . . to the service of the folk’.^71^ In this context, we can again see how despite professing an informal and progressive educational outlook, the Folk drew upon techniques and materialities associated with formal education – here using certificates, exams and inspections to judge prospective volunteers.

The Folk stated in the introduction to their course material that ‘the diploma will be one which we hope all Leaders will strive to obtain so that a degree of uniformity in our Folk training of leaders will give us a secure foundation on which to build up our more advanced educational work’.^72^ Through looking at marked exam scripts and the subjective comments of assessors (also volunteers), it appears there was a high standard set for trainee helpers, rather than this process being a tokenistic exercise. For example, in a failed exam, an assessor stated ‘I recommend the candidate fail the exam, but be congratulated on a very good try . . . [w]ith one more year Folk knowledge, will easily pass next year’ whilst another ‘trainee’ who passed still had comments that the ‘candidate is capable of going a bit deeper’.^73^ This highlights the subjective nature of required standards for unpaid volunteers and the regulation of adults within the organization, which I suggest became more acute in the 1960s and 1970s. In 1966, the Folk admitted that whilst in the early years they had ‘accepted almost any kind of applicant who has been prepared “to have a go”’,^74^ the issue of adult training needed to be further enhanced and standardized. In the same telling report, the Folk stated that ‘[w]e cannot justify our claim for educational grants if our work is sub-standard’.^75^ Indeed, the organization was eligible for a series of educational grants and financial support in the mid-20th century beyond its charitable fundraising, and so although informal, alternative, and radical – the Folk were still engaging with the mechanisms and finances of the state in terms of an emerging and increasingly professionalized youth work landscape that need well-*educated –* or more accurately – well-*trained* volunteers.^76^

The final part of this section’s discussion focuses on age and volunteering. The Folk often stated they ‘welcome[d] all who are young in spirit under its banner’^77^ with seemingly no upper age limit for adult volunteers (as was the case for many years in scouting). In valuing a sense of ‘youthfulness’, the Folk drew on a series of idealized constructions about childhood as carefree, wonderful and pure, whilst suggesting that these were desired attributes in their responsible adult leaders.^78^ However, young people were also seen as possessing leadership capabilities and in some cases completed adult training. Of the 56 candidates who sat the leaders’ diploma examination in 1946, 38 were under 18 years old, with the youngest just 13.^79^ At first glance this is surprising, and yet it does chime with the Folk’s wider aims surrounding youth, responsibility and citizenship. As early as 1931, Folk elders stated that ‘among the youth of our movement are many who can lead, if they will first learn’.^80^ Learning was therefore seen as the prelude to leadership, a necessary foundation before the voluntary work of the Folk could be undertaken. Further analysis, however, shows that only 17 of those 38 individuals under 18 who sat the leaders’ examination that year passed, and over half of these successful applicants were aged 17.^81^ On one hand, it is striking that young people in their early teens were allowed to sit an ‘adult’ examination – the result of which could have enabled them to formally run local Groups, and yet on the other hand, the very fact an exam, marking criteria, and regulations surrounding this ‘vocation’ existed demonstrates a clear understanding from Folk elders that standards and knowledge needed to be enforced to safeguard this process before ‘adult’ responsibilities began. It should still be noted that three 15-year-olds passed the leader’s diploma in 1946. Age was therefore not a barrier to adult leadership within the organization *if* the necessary standards were achieved; ‘adulthood’ within this institutional space related to a required standard of knowledge and aptitude, rather than biological characteristics. This also reflects how notions of childhood and adulthood have shifted over time and are historically specific.^82^ Overall, the Folk was therefore a complex and contradictory space: cementing age boundaries in their membership criteria for young people, yet ‘giving a chance’ to its children to ‘make good’ as potential ‘adult’ volunteers.^83^

Overall, the discussion in this article has illustrated that training was at the heart of the Folk’s activities – whether for children, young people or adults. Indeed, the Folk’s business as a youth organization was not just *political*, but as Shada stressed in that early committee meeting, *educational*. Clearly though, the two objectives were intertwined in an approach by the organization to train youth for a politically just life. It is important to highlight ‘the political’ here in terms of training and debates on neoliberalism. Throughout the article, examples of tensions between the ideology and practice of the Woodcraft Folk have been presented, some of which can be understood in terms of internal struggles over the organization’s identity and connections to ‘radical’ politics while at the same time striving to be popular and utilize more conventional educational ideas. The very notion of ‘training’ has important political dimensions that whilst often benign, can also be deeply troubling.^84^ In a contemporary context, there is great scope to research the relationship between youth organizations and informal education in the context of neoliberal agendas and diverse articulations of ‘the political’.

## Conclusion

This article has presented an enlivened historical geography of education and voluntarism within one British youth movement and crafted an argument about the training of youth *and* adults within this institutional space over time. Through a unique historical approach to the geographies of education, this article has also connected with wider debates on the geographies of voluntarism and children, youth and families. I have argued that the rationale and activities through which the Woodcraft Folk trained young people and adults illustrates how this youth organization was a complex assemblage of learners, spaces and practices, at times blurring the boundaries between formal and informal education. This paper therefore highlights the value of a cultural approach to the geographies of education, exploring the performances of (informal) education in and through space, as well as contributing a real focus on *training* spaces within the ‘disciplinary endeavour’ on the geographies of education.^85^ In showing that this organization had a wider spatial remit and more complex institutional geographies of education across the lifecourse than have been acknowledged in the historical literature, this article also speaks to those beyond geography, specifically historians of voluntary action, childhood and civil society. In this final concluding section, I want to reflect more broadly on the wider relevance of this study for geographers and suggest some further avenues for future inter-disciplinary research.

During the first 50 years of its activities as a youth organization, the Folk called for reforms to state education, whilst at the same time utilizing school-based techniques and materials; championed the freedom and energy of its voluntary base, yet required them to sit formal exams and hand-in their notebooks for inspection; and finally, claimed to be driven by the interests of the child, yet constructed an educational programme with a central aim to train young people on the road to citizenship. At times contradictory, the example of the Woodcraft Folk between 1925–75 illustrates the complex and often fluid relationship between formal and informal learning and the subsequent challenges for researchers in defining certain educational spaces as ‘formal’ or ‘informal’. This article’s central contribution has therefore been to illustrate that relationship as part of a wider argument on the importance of recognizing training spaces within research on the geographies of education. I would argue that there is great potential for the complex geographies of training to be explored more fully across a range of sites and settings; for example, through studies on internships, apprenticeships, entrepreneurship activities, adult education, and youth and community work.

This article has also highlighted some important connections between education and volunteering. This relationship has shifted over time and relates to both formal and informal spaces of education. As Hardill and Baines recently stated, volunteering in the United Kingdom under New Labour was strongly linked to education; for example, one component of the General Certification in Secondary Education (GCSE) in ‘Citizenship’ was ‘evidence of the candidate’s lived experience of volunteering’.^86^ What this present article has illustrated is that notions of qualifications in the voluntary sector are not new and that diplomas, courses, training events and assessed performances were part and parcel of the volunteering culture within certainly this (and anecdotally other) youth organizations in the early to mid-20th century. The need for geographers and other researchers to interrogate the connections between education, volunteering and civil society is pressing. Volunteers have been positioned as a panacea to address the gaps the state has left through funding withdrawals in a range of historical and contemporary contexts.^87^ These funding cuts have often been felt most acutely in youth service provision, and therefore a deeper understanding of the relationship between the state, civil society and youth is crucial, particularly as childhood is often mobilized in times of national and global change and anxieties.^88^ Overall, this youth organization championed volunteering as a worthwhile activity to potential helpers, often positioning it as an antidote to popular culture and other leisure pursuits. For example, in a set of minutes from a Woodcraft Folk conference held at Weymouth in April 1965, one volunteer is recorded as stating that ‘on his way home he often looked in at windows and saw people watching television and then thought what a good job he was doing spending his time on the work of the Folk. He stayed in the movement because he enjoyed doing this work with kids . . . he told people it was not a hardship – he enjoyed doing it’.^89^ This brief insight into the culture of volunteering could prove a fruitful avenue for future research. Indeed, there is scope for local oral history projects to further interrogate some of the enlivened historical geographies of voluntarism in these, and other, organizations, as well as the wider role of parents and families in creating, maintaining and negotiating these institutional spaces. This focus would also connect to debates on age and the lifecourse. Here, I illustrated that whilst imagined as a space *for young people* run *by adults* – the realities of the Folk were more complex, with young teenagers sitting adult exams to become official volunteers, and adults encouraged to be more ‘youthful’. This highlights some of the continued challenges in children’s geographies and beyond surrounding age and the liminal period of youth.^90^ I would therefore suggest that a cultural-historical approach could offer some useful methodological insights on age to compliment work on these themes in social geography. Indeed, there is a real focus in historical studies at present on parenting, childhood and age across diverse historical epochs and geographical contexts^91^ and there could be a potentially useful dialogue between geographers and historians on critical debates surrounding age, families and the lifecourse.

## Notes

1. For example, J. Springhall, *Youth, Empire and Society: British Youth Movements 1883–1940* (Beckenham: Croom Helm, 1977); T. Proctor *On My Honour: Guides and Scouts in Interwar Britain* (Philadelphia, PA: American Philosophical Society, 2002).

2. For example, T. Ploszajska, ‘Moral Landscapes and Manipulated Spaces: Gender, Class and Space in Victorian Reformatory Schools’, *Journal of Historical Geography*, 20(4), 1994, pp. 413–29; T. Ploszajska, ‘Constructing the Subject: Geographical Models in English Schools, 1870-1944’, *Journal of Historical Geography*, 22(4), 1996, pp. 388–98; E.A. Gagen, ‘Making America Flesh: Physicality and Nationhood in Early Twentieth Century Physical Education Reform’, *cultural geographies*, 11(4), 2004, pp. 417–42.

3. C. Hanson Thiem, ‘Thinking Through Education: The Geographies of Contemporary Educational Restructuring’, *Progress in Human Geography*, 33(2), 2009, pp. 154–73; S.L. Holloway, P. Hubbard, H. Jöns and H. Pimlott–Wilson, ‘Geographies of Education and the Significance of Children, Youth and Families’, *Progress in Human Geography*, 34(5), 2010, pp. 583–600; S.L. Holloway and H. Jöns, ‘Geographies of Education and Learning’, *Transactions of the Institute of British Geographers*, 37(4), 2012, pp. 482–8.

4. S.L. Holloway and G. Valentine (eds), *Children’s Geographies: Playing, Living, Learning* (London: Routledge, 2000); L. Holt (ed.), *Geographies of Children, Youth and Families: An International Perspective* (Oxon and New York: Routledge, 2011).

5. On these themes, see E. Wainwright, E. Marandet, S. Buckingham and F. Smith, ‘The Training–to–Work Trajectory: Pressures for and Subversions to Participation in the Neoliberal Learning Market in the UK’, *Gender, Place and Culture: A Journal of Feminist Geography*, 18(5), 2011, pp. 635–54; C. Philo, H. Parr and N. Burns, ‘“An oasis for us”: “In-between” Spaces of Training for People with Mental Health Problems in the Scottish Highlands’, *Geoforum*, 36(6), 2005, pp. 778–91.

6. See P. Jackson, ‘Rematerializing Social and Cultural Geography’, *Social and Cultural Geography*, 1(1), 2000, pp. 9–14; T. Cresswell, ‘New Cultural Geography – an Unfinished Project?’, *cultural geographies*, 17(2), 2010, pp. 169–74; D.P. Smith, K. Browne and D. Bissell, ‘Reinvigorating Social Geographies? A “Social Re/turn” for a Changing Social World: (Re)opening a Debate’, *Social & Cultural Geography*, 12(6), 2011, pp. 517–28, on this much wider debate.

7. For example, P. Kraftl, *Geographies of Alternative Education: Diverse Learning Spaces for Children and Young People* (Bristol: Policy Press, 2013).

8. P. Gruffudd, ‘The Countryside as Educator: Schools, Rurality and Citizenship in Inter-War Wales’, *Journal of Historical Geography*, 22(4), 1996, pp. 412–23; A. Maddrell, ‘Empire, Emigration and School Geography: Changing Discourses of Imperial Citizenship, 1880-1925’, *Journal of Historical Geography*, 22(4), 1996, pp. 373–87; Ploszajska, ‘Constructing the Subject’; T. Ploszajska, ‘Down to Earth? Geography Fieldwork in English Schools, 1870-1944’, *Environment and Planning D: Society and Space*, 16(6), 1998, pp. 757–74.

9. Ploszajska, ‘Moral Landscapes’; Gagen, ‘Making America Flesh’; E.A. Gagen, ‘An Example to Us All: Child Development and Identity Construction in Early 20th-Century Playgrounds’, *Environment and Planning A*, 32(4), 2000, pp. 599–616.

10. Holloway and Jöns, ‘Geographies of Education and Learning’, p. 486.

11. See C. Philo and H. Parr, ‘Institutional Geographies: Introductory Remarks’, *Geoforum*, 31(4), 2000, pp. 513–21.

12. P. Hopkins and R. Pain, ‘Geographies of Age: Thinking Relationally’, *Area*, 39(3), 2007, pp. 287–94.

13. See R. Vanderbeck, ‘Intergenerational Geographies: Age Relations, Segregation and Reengagements’, *Geography Compass*, 1(2), 2007, pp. 200–21.

14. Holloway and Jöns, ‘Geographies of Education and Learning’, p. 482. See also Hanson Thiem, ‘Thinking Through Education’; S. Mills and P. Kraftl, *Informal Education, Childhood and Youth: Geographies, Histories, Practices* (Basingstoke: Palgrave Macmillan, 2014)

15. C. Milligan, ‘Geographies of Voluntarism: Mapping the Terrain’, *Geography Compass*, 1(2), 2007, pp. 183–99; I. Hardill and S. Baines, *Enterprising Care? Unpaid Voluntary Action in the 21^st^ Century* (Bristol: Policy Press, 2011).

16. S.L. Holloway, ‘Mother and Worker? The Negotiation of Motherhood and Paid Employment in Two Urban Neighbourhoods’, *Urban Geography*, 20(5), 1999, pp. 438–60; E. Wainwright and E. Marandet, ‘Geographies of Family Learning and Aspirations of Belonging’, *Children’s Geographies*, 9(1), 2011, pp. 95–109.

17. Hopkins and Pain, ‘Geographies of Age’.

18. Philo et al., ‘“An oasis for us”’; Wainwright et al., ‘The Training–to–Work Trajectory’.

19. S. Buckingham, E. Marandet, F. Smith, E. Wainwright and M. Diosi, ‘The Liminality of Training Spaces: Places of Private/Public Transitions’, *Geoforum*, 37(6), 2006, pp. 895–905; K. Mitchell, ‘Neoliberal Governmentality in the European Union: Education, Training and Technologies of Citizenship’, *Environment and Planning D: Society and Space*, 24(3), 2006, pp. 389–407; Wainwright and Marandet, ‘Geographies of Family Learning’.

20. S. Malik, ‘Graduate’s Poundland Victory Leaves Government Work Schemes in Tatters’, *The Guardian*, 12 February 2013, <http://www.guardian.co.uk/society/2013/feb/12/graduate–poundland–government–work–schemes> (11 October 2013).

21. Philo et al., ‘“An oasis for us”’;

22. S. Fielding, ‘Walk on the Left: Children’s Geographies and the Primary School’, in S.L. Holloway and G. Valentine (eds), *Children’s Geographies: Playing, Living, Learning* (London: Routledge, 2000).

23. D. Heater, ‘The History of Citizenship Education in England’, *The Curriculum Journal*, 12(1), 2001, pp. 103–23; J. Pykett, ‘Making Citizens in the Classroom – An Urban Geography of Citizenship Education?’, *Urban Studies*, 46(4), 2009, pp. 803–23.

24. S. Mills, ‘“An Instruction in Good Citizenship”: Scouting and the Historical Geographies of Citizenship Education’, *Transactions of the Institute of British Geographers*, 38, 2013, pp. 120–34.

25. T. Jeffs and M. Smith, *Informal Education: Conversation, Democracy and Learning* (London: Education Now Publishing Co–operative, 2005); Mills and Kraftl, *Informal Education, Childhood and Youth*.

26. For example, D. Conradson, ‘Doing Organisational Space: Practices of Voluntary Welfare in the City’, *Environment and Planning A*, 35, 2003, pp. 1975–92; N. Fyfe and C. Milligan, ‘Out of the Shadows: Exploring Contemporary Geographies of the Welfare Voluntary Sector’, *Progress in Human Geography*, 27, 2003, pp. 397–413; C. Milligan and D. Conradson (eds), *Landscapes of Voluntarism: New Spaces of Health, Welfare and Governance* (Bristol: Policy Press, 2006).

27. M. Baillie Smith, N. Laurie, P. Hopkins and E. Olson, ‘International Volunteering, Faith and Subjectivity: Negotiating Cosmopolitanism, Citizenship and Development’, *Geoforum*, 45, 2013, pp. 126–35.

28. C. Holdsworth, ‘Why Volunteer? Understanding Motivations for Student Volunteering’, *British Journal of Educational Studies*, 58(4), 2010, pp. 421–37.

29. F.M. Smith, H. Timbrell, M. Woolvin, S. Muirhead and N. Fyfe, ‘Enlivened Geographies of Volunteering: Situated, Embodied and Emotional Practices of Voluntary Action’, *Scottish Geographical Journal*, 126(4), 2010, pp. 258–74 (p. 258).

30. M. Oppenheimer and N. Deakin (eds), *Beveridge and Voluntary Action in Britain and the Wider British World* (Manchester: Manchester University Press, 2011); M. Hilton and J. McKay (eds), *The Ages of Voluntarism: How we got to the Big Society* (Oxford: Oxford University Press, 2012).

31. G. Valentine, ‘Boundary Crossings: Transitions from Childhood to Adulthood’, *Children’s Geographies*, 1(1), 2003, pp. 37–52.

32. Holloway, ‘Mother and Worker?’; S.L. Holloway, ‘Local Childcare Cultures: Moral Geographies of Mothering and the Social Organisation of Pre–School Education’, *Gender, Place and Culture: A Journal of Feminist Geography*, 5(1), 1998, pp. 29–53.

33. S. Aitken, ‘Fathering and Faltering: “Sorry, but you don’t have the necessary accoutrements”’, *Environment and Planning A*, 32(4), 2000, pp. 581–98.

34. A. Henderson, *Insights from the Playgroup Movement: Equality and Autonomy in a Voluntary Organisation* (Stoke-on-Trent: Trentham Books, 2011).

35. E. Jupp, ‘The Feeling of Participation: Everyday Spaces and Urban Change’, *Geoforum*, 39(1), 2008, pp. 311–43.

36. S.L. Holloway and H. Pimlott-Wilson, ‘Enriching Children, Institutionalizing Childhood? Geographies of Play, Extra-curricular Activities, and Parenting in England’, *Annals of the Association of American Geographers*, 104(3), 2014, pp. 613–27.

37. Hopkins and Pain, ‘Geographies of Age’.

38. Mills, ‘An Instruction in Good Citizenship’, p. 123.

39. 1935, WF/1/2.

40. Springhall, *Youth, Empire and Society*; D. Prynn, ‘The Woodcraft Folk and the Labour Movement 1925-70’, *Journal of Contemporary History*, 18, 1983, pp. 79–95; M. Davis, *Fashioning a New World: A History of the Woodcraft Folk* (Loughborough: Holyoake Books, 2000).

41. 1928, WF/1/1.

42. See S. Mills ‘“A Powerful Educational Instrument”: The Woodcraft Folk, Indoor/Outdoor Nature and Pedagogical Practice 1925-1975’, in S. Mills and P. Kraftl (eds), *Informal Education, Childhood and Youth: Geographies, Histories, Practices* (Basingstoke: Palgrave Macmillan, 2014).

43. 1930, WF/2/5.

44. Davis, 2000, 123.

45. It should be noted that these age ranges and names of sections varied throughout the period studied. These relate to the most commonly used in the mid-20th century.

46. 1947, WF/1/4A.

47. 1949, WF/6/50, emphasis added.

48. On wider debates surrounding youth citizenship, see L. Staeheli, ‘Political Geography: Where’s Citizenship?’, *Progress in Human Geography*, 35(3), 2011, pp. 393–400; T. Skelton, ‘Children, Young People and Politics: Transformative Politics for a Discipline?’, *Geoforum*, 49, 2013, pp. R4–R6.

49. See, for example, J. Ash, M. Paterson and J. Pykett, ‘Plasticities: The Place of Critical Neuroscience, Materialities and Behaviors’, co–organized session at *The Annual Conference of the Royal Geographical Society with Institute of British Geographers*, London, August 2011.

50. WF/1/1.

51. 1937, WF/1/2, original emphasis.

52. 1928, WF/1/1, emphasis added.

53. 1930, WF/2/5.

54. 1935, WF/11/125.

55. 1948, WF/1-4A.

56. 1949, WF/6/50.

57. WF/6/50.

58. c.1930, WF/2/5.

59. 1930, WF/2/5.

60. 1949, WF/6/50.

61. J. English, ‘Empire Day in Britain 1904–1958’, *The Historical Journal*, 49(1), 2006, pp. 247–76.

62. 1933, WF/1/1, emphasis added.

63. 1930, WF/2/5, emphasis added.

64. 1936, WF/11/125, emphasis added.

65. Kraftl, *Geographies of Alternative Education*.

66. Philo and Parr, ‘Institutional Geographies’.

67. WF/7/51.

68. L. Paul, *The Republic of Children: A Handbook for Teachers of Working Class Children* (London: Allen & Unwin, 1938).

69. Gagen, ‘Making America Flesh’.

70. c.1940s, WF/7/52.

71. c.1940s, WF/7/52.

72. c.1940s, WF/7/53A.

73. 1955, WF/7/77B.

74. 1966, WF/10/104.

75. 1966, WF/10/104.

76. B. Davies, *From Voluntaryism to Welfare State: A History of the Youth Service in England Vol. 1: 1939–1979* (Leicester: Youth Work Press, 1999); G. Verschelden, F. Coussée, T. Van de Walle and H. Williamson (eds), *The History of Youth Work in Europe – Relevance for Today’s Youth Work Policy – Volume 1* (Council of Europe publishing, 2009).

77. 1930, WF/2/5.

78. On notions of childhood, see Holloway and Valentine, *Children’s Geographies*; C. Jenks *Childhood* (London: Routledge, 1996).

79. 1946, WF/7/55.

80. 1931, WF/1/1.

81. 1946, WF/7/55.

82. See P. Ariès, *Centuries of Childhood: A Social History of Family Life* (New York: Vintage Books, 1962).

83. 1940, WF/1/3.

84. See Mills, ‘An Instruction in Good Citizenship’.

85. Holloway and Jöns, ‘Geographies of Education and Learning’.

86. Hardill and Baines, *Enterprising Care?*, p. 36.

87. Hilton and McKay, *The Ages of Voluntarism*.

88. C. Katz, ‘Childhood as Spectacle: Relays of Anxiety and the Reconfiguration of the Child’, *cultural geographies*, 15(1), 2008, pp. 5–17.

89. WF/2/21.

90. Valentine, ‘Boundary Crossings’.

91. For example, J. Bailey, *Parenting in England 1760-1830: Emotion, Identity and Generation* (Oxford: Oxford University Press, 2012); P. Thane and T. Evans, *Sinners? Scroungers? Saints? Unmarried Motherhood in Twentieth-century England* (Oxford: Oxford University Press, 2012).

